# Predictors of Change in Bodily Pain in Early Rheumatoid Arthritis: An Inception Cohort Study

**DOI:** 10.1002/acr.21723

**Published:** 2012-09-28

**Authors:** Daniel F McWilliams, Weiya Zhang, Josephine S Mansell, Patrick D W Kiely, Adam Young, David A Walsh

**Affiliations:** 1 Arthritis Research UK Pain Centre, University of NottinghamNottingham, UK; 2 St. George's Healthcare NHS TrustLondon, UK; 3 West Hertfordshire Hospitals NHS Trust, St. AlbansUK; 4 Arthritis Research UK Pain Centre, University of Nottingham, Nottingham, and Sherwood Forest Hospitals NHS Foundation TrustSutton-in-Ashfield, UK

## Abstract

**Objective:**

To investigate possible predictors for lack of pain improvement after 1 year of treatment for early rheumatoid arthritis (RA).

**Methods:**

The Early Rheumatoid Arthritis Network (ERAN) database was used for analysis of baseline and 1-year pain data. The ERAN is a hospital-based inception cohort of 1,189 people. Short Form 36 questionnaire bodily pain scores were used to calculate change in pain at 1 year as the outcome. The proportion of the Disease Activity Score in 28 joints (DAS28) attributable to patient-reported components (joint tenderness and visual analog scale score; DAS28-P) at baseline was derived as a predictor. Predictors of less improvement in pain were investigated using adjusted odds ratios (OR_adj_) generated by logistic regression, adjusting for 14 additional clinical and demographic covariates.

**Results:**

Greater pain at baseline was associated with sex, high DAS28, worse mental health, and smoking. Most patients with early RA reported incomplete improvement in bodily pain after 1 year. The DAS28-P index did not significantly change in the patients whose disease remained active. Less improvement in pain was predicted by female sex (OR_adj_ 3.41, 95% confidence interval [95% CI] 1.35–8.64) and a high DAS28-P index at baseline (OR_adj_ for tertiles 2.09, 95% CI 1.24–3.55). Other conventional RA risk factors did not predict pain changes.

**Conclusion:**

The factors most likely to predict less improvement in pain in early RA are female sex and a high DAS28-P index. A high DAS28-P index may reflect greater contributions of noninflammatory factors, such as central sensitization, to pain. Strategies in addition to inflammatory disease suppression may be required to adequately treat pain.

## INTRODUCTION

The progress of rheumatoid arthritis (RA) is variable, and despite the potential for severe disabling disease, many patients will enter remission. Indeed, remission is becoming more common (1) and achieving it quickly may improve long-term outcomes (2). Recent clinical trials show success in treating early RA aggressively (3), and also when treatment choice is explicitly driven by regular examinations for disease activity (4).

The Disease Activity Score in 28 joints (DAS28) is commonly used to inform treatment decisions (5). The DAS28 is a composite score calculated from 28 tender joint counts (TJCs), 28 swollen joint counts (SJCs), acute-phase response (often the erythrocyte sedimentation rate [ESR]), and a visual analog scale (VAS) of the patient's self-reported disease activity (general health [GH]) (6, 7). Of these factors, swollen joints and acute-phase response are determined by the assessor, whereas the VAS and tender joints are reported by the patient, the latter in response to pressure applied to the joint (8).

High DAS28 scores influence the choice of treatments of early RA, and it is therefore important to understand the relationship between DAS28 and inflammatory disease activity, as well as potential effects of confounding factors. Although self-reported disease activity and tenderness on palpation increase alongside inflammatory disease activity, they may also be increased by changes in pain processing such as central sensitization or by comorbidities. Patients without RA who have fibromyalgia or central sensitization report high disease activity and tenderness, generating high DAS28 scores despite low SJC and acute-phase response that may be comparable to those of people with active RA (9). Evidence of central sensitization and joint inflammation may coincide, thereby confounding interpretation of the DAS28 in people with RA (10). People with concurrent RA and fibromyalgia report increased pain (11) and show higher DAS28 scores (12) than those with RA alone, perhaps indicating that central sensitization or painful comorbidity influences the scoring.

Opinions differ as to whether fibromyalgia should be seen as a comorbidity in RA, or whether the clinical features of fibromyalgia represent changes in pain processing associated with the arthritis. Seeing fibromyalgia as a comorbidity suggests potential for treatments directed at mechanisms other than joint inflammation in a subgroup of patients. The extent to which pain processing is aberrant in RA may, however, occur across a continuum. Application of fibromyalgia classification criteria in RA may identify a subgroup of patients with the most abnormal pain processing (13), but also may conceal a larger number of patients in whom similar pain mechanisms make an important contribution to their symptoms. In clinical practice, the DAS28 is often interpreted with regard to the contribution of TJC and GH to the total score, based on individual clinical judgment rather than standardized or quantifiable criteria. We propose that a derived measure for the contribution of these patient-reported components to DAS28, measured here as the DAS28-P index, may be a convenient and useful index of noninflammatory pain mechanisms in RA.

Pain is often the most bothersome symptom of RA (14), but epidemiologic risk factors for worse pain in RA have been researched less thoroughly than outcomes such as disease activity and radiologic damage. Pain is associated with high disease activity (15) and can be reduced by early effective treatment of inflammatory disease (16). Female sex (15, 17) may be related to worse pain over time, and psychological factors influence pain reporting in RA (15, 18). Radiographic changes may be linked to future pain in people with RA (15). Furthermore, changes in pain processing through peripheral and/or central sensitization may contribute to pain in RA (10, 19, 20).

The aims of this study were to determine if well-known RA risk factors are associated with pain changes during the first year after presentation with RA, and also if the contribution of patient-reported components to the DAS28 at baseline predicts pain outcome. We used data from people recruited to the Early Rheumatoid Arthritis Network (ERAN) (21, 22), an inception cohort study of patients with newly diagnosed RA receiving standard care.

**Significance & Innovations**

- A derived patient-reported outcome measure for the proportion of the Disease Activity Score in 28 joints (DAS28) contributed by tender joint count and general health (DAS28-P) may help identify noninflammatory influences on pain.
- A high DAS28-P at baseline predicts less improvement in pain at 1 year in patients with rheumatoid arthritis, despite traditional disease-modifying therapy.

## PATIENTS AND METHODS

### Patients

The ERAN inception cohort study (21, 22) recruits from 22 outpatient centers in the UK and Ireland (22, 23). Recruitment began in April 2002, and still continues with followup. Prospectively collected data used in this study were retrieved during winter 2010, and included all patients who had attended the relevant clinics and provided appropriate data. Previously reported data from the ERAN include examinations of the patterns of care and disease outcomes (24, 25), validation of work questionnaires (26, 27), and factors influencing choice of therapy (5). Patients are recruited to the ERAN following a physician diagnosis of RA. ERAN centers monitor and treat patients according to local practice, without requirement to follow any particular treatment protocol. Most of the patients recruited during this period were treated initially with sulfasalazine or methotrexate as monotherapy (5). At the time of this study, data from 1,189 patients were available for analysis. The study was approved by the Trent Research Ethics Committee (ref. 01/4/047) and all participants gave signed informed consent in line with the Declaration of Helsinki.

### Data collection

Data collected prospectively at baseline and at 1-year followup were used in this study. A clinical examination and interview were performed at each visit that were used to derive the DAS28-ESR, determine the number of 1987 American College of Rheumatology (ACR) RA diagnostic criteria that were fulfilled (28), and record extraarticular disease manifestations or comorbidities (coded as International Statistical Classification of Diseases and Related Health Problems, Tenth Revision chapters on clinical record forms and supplemented by free text). The presence of erosions was documented from radiographs of the hands and feet that were taken for clinical reasons. Demographic and clinical data were obtained from case notes and a clinical interview and participants were invited to complete the Short Form 36 (SF-36) questionnaire (29) and the Health Assessment Questionnaire (HAQ) (30). Results of clinical tests for ESR and rheumatoid factor (RF) were obtained from the clinical record. Seropositive individuals were those classified as positive for RF or antibodies to citrullinated peptide. Results reported as negative or weakly positive for RF according to local reference ranges were classified as seronegative.

All observations were recorded and stored at the outpatient centers and were also forwarded to the central database. The verification of local data was performed at visits by the study coordinator (5). All assessments were timed to coincide with clinical visits, such that 1-year followup data in this report represent data collected 9–18 months after baseline assessment (median 370 [interquartile range (IQR) 350–408] days after baseline).

### SF-36 subscales

The SF-36 Health Survey for patient-based assessment of quality of life was used to evaluate aspects of health that are frequently affected by disease (29). The SF-36 is well accepted for assessing quality of life in a number of disease populations, including RA (31, 32). We used the SF-36 bodily pain score as a measure of pain in people with early RA. For every subscale used (bodily pain, mental health, vitality, and physical function), the questionnaire answers were transformed into a 0–100 range, as per guidelines (29), but were not normed for age or sex (since both would be adjusted for during analysis). Each raw subscale is included in its unaltered form, and this was also used to calculate the percentage changes between baseline and 1 year. Missing values for vitality and mental health were replaced with the mean response if sufficient questions were answered (29). Answers from both version 1 and version 2 of the SF-36 questionnaire were included, and subscale calculations were modified where necessary. Cases with any missing data for the bodily pain outcome variable were excluded (n = 26 at baseline). Because thresholds for pain scores are difficult to establish and interpret, the percentage change in bodily pain score from baseline to 1 year was calculated for longitudinal analyses. Participants with less improvement were defined as those with below median pain change.

### DAS28 and DAS28-P index

The DAS28 score was calculated using the ESR and according to standard formulae (8). Additionally, for patients with active disease (DAS28 ≥3.2), the fraction of the total DAS28 score contributed by patient-reported components (TJC and patient global assessment) was calculated and is referred to as the DAS28-P index. Those with a DAS28 <3.2 did not have the DAS28-P index calculated in order to reduce the variation derived from calculations with small denominators (DAS28-P index calculated for n = 826 at baseline and n = 476 at 1 year; see Supplementary Appendix A, available in the online version of this article at http://onlinelibrary.wiley.com/journal/10.1002/(ISSN)2151-4658).

The DAS28-P index is calculated as follows:


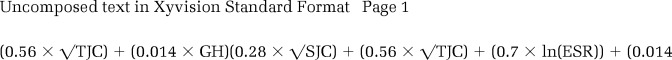


### Statistical analysis

The measures associated with the DAS28-P index were analyzed using Spearman's correlations and Mann-Whitney U tests, with multiple regression being used to adjust for confounding. Confirmatory secondary analyses of selected 1-year data were performed using Wilcoxon's paired tests.

For measures associated with pain and pain changes, medians and IQRs or percentages are shown throughout. DAS28 scores were classified into European League Against Rheumatism activity groups (where 0–3.19 = inactive, 3.2–5.19 = active, and ≥5.2 = severe) (33), body mass index (BMI) was classified into World Health Organization groups (where <25 kg/m^2^ = normal, 25–29.9 kg/m^2^ = overweight, and ≥30 kg/m^2^ = obese) (34), and other continuous variables were split into tertiles of increasing severity or magnitude. Odds ratios and 95% confidence intervals (95% CIs) were calculated initially without adjustment and compared to the least severe reference group/tertile.

Logistic regression analyses were performed to assess associations with greater than median bodily pain or less than median change in bodily pain at 1 year (less improvement). Data on nonsteroidal antiinflammatory drug (NSAID), disease-modifying antirheumatic drug (DMARD), and regular corticosteroid usage were included in the models to address possible confounding by treatment of inflammatory disease. Secondary analyses with smaller numbers of baseline variables and using the absolute pain score at 1 year were undertaken to test the robustness of findings from full logistic regression models. Statistical analysis was performed using SPSS, version 14 (IBM). Statistical significance was taken to be *P* values less than 0.05 and when 95% CIs did not encompass unity.

## RESULTS

### Demographics

Of the 1,189 patients recruited to the database by the time of the study, 977 and 609 had SF-36 bodily pain scores available for baseline and 1 year, respectively. The demographic characteristics of the study population and the study groups are shown in Table 1, and the characteristics of those with SF-36 data available were similar to the entire study population. As previously published, the majority of patients in the ERAN received DMARD monotherapy during the first year (25), and in our subset we found that 56% received methotrexate and 40% received sulfasalazine as part of their treatment regimens at the 1-year time point.

**Table 1:** Demographic characteristics*

	ERAN database (n = 1,189)	Baseline SF-36 score (n = 977)	1-year SF-36 score (n = 609)
Age, median (IQR) years	58 (47–68)	58 (48–67)	58 (48–67)
BMI, median (IQR) kg/m^2^	26.8 (23.9–30.3)	26.8 (23.9–30.3)	26.9 (24.1–30.2)
Women, % (no./total)	68 (805/1,189)	68 (660/977)	66 (404/609)
White, % (no./total)	94 (1,116/1,188)	97 (947/977)	98 (594/609)
Current smoker, % (no./total)	33 (388/1,166)	34 (327/975)	31 (191/608)

* The demographic characteristics of the Early Rheumatoid Arthritis Network (ERAN) study population and those with Short Form 36 (SF-36) data available were statistically similar. IQR = interquartile range; BMI = body mass index.

### DAS28-P index

The baseline median DAS28-P index was 0.43 (IQR 0.36–0.49). The DAS28-P was positively correlated with the DAS28, but less so than the individual components from which both scores were derived (Table 2). In order to determine the possible clinical relevance of the DAS28-P index, associations with other characteristics related to the patient experience of disease were investigated. At baseline, the DAS28-P index was associated with worse bodily pain scores (r = −0.31, *P* < 0.001), higher DAS28 (r = 0.34, *P* < 0.001), greater disability measured by the HAQ (r = 0.28, *P* < 0.001), and younger age (r = −0.22, *P* < 0.001). Baseline scores on the SF-36 subscales indicating lower vitality (r = −0.26, *P* < 0.001) and physical function (r = −0.20, *P* < 0.001) were also associated with a higher DAS28-P index. Multiple linear regression was performed to investigate which patient-based factors were associated with the DAS28-P index at baseline, while controlling for other confounders. Subscales of the SF-36, HAQ, and DAS28 were included with symptom duration, serology, 1987 ACR criteria, comorbidities, extraarticular disease, smoking history, age, sex, and BMI as covariates. After adjustment for other variables, the DAS28-P index was significantly associated with worse bodily pain, higher DAS28 score, and younger age (β = −0.18, *P* = 0.003; β = 0.25, *P* < 0.001; and β = −0.19, *P* < 0.001, respectively).

**Table 2:** Relationships between the DAS28 and its individual score components and the DAS28-P*

	DAS28
DAS28-P	0.34 (0.28–0.40)
TJC	0.83 (0.81–0.85)
SJC	0.72 (0.69–0.75)
ESR	0.62 (0.58–0.66)
VAS GH	0.66 (0.63–0.70)

* Spearman's correlation coefficients (95% confidence intervals) with the Disease Activity Score in 28 joints (DAS28) show the proportion of the DAS28 attributable to patient-reported components (DAS28-P) and individual components of the DAS28. The DAS28-P shows less dependence upon the DAS28 than the individual components. *P* < 0.01 for all components. TJC = tender joint count; SJC = swollen joint count; ESR = erythrocyte sedimentation rate; VAS = visual analog scale; GH = general health.

The median bodily pain scores improved from 41 (IQR 22–62) at baseline to 51 (IQR 31–74) at the 1-year followup (Z = −7.6, *P* < 0.001). Mean ± SD total DAS28 scores also showed a statistically significant improvement from baseline (4.8 ± 1.6) to 1 year (3.8 ± 1.6; t = 14.3, *P* < 0.001). The association between higher DAS28-P index and greater bodily pain was retained at both time points (baseline: r = −0.31, *P* < 0.001; 1 year: r = −0.45, *P* < 0.001). The DAS28-P, calculated for those with active disease, was similar at 1 year (median 0.45, IQR 0.35–0.52) and at baseline (median 0.43, IQR 0.36–0.49; Z = −0.62, *P* = 0.534), despite a significant decrease in the total DAS28 between baseline (median 5.6, IQR 4.6–6.3) and 1 year (median 4.7, IQR 4.0–5.6) in this subgroup (*P* < 0.001).

### Baseline associations with pain

When baseline only was considered, logistic regression analysis revealed that several covariates were associated with baseline pain score (Table 3). Worse pain at baseline was observed in men and those patients with shorter disease duration, high DAS28, high DAS28-P index, SF-36 scores indicating poorer mental health, and previous smoking.

**Table 3:** Baseline associations of pain and classic risk factors for RA severity*

Covariate and tertiles/groups	Unadjusted analyses		Logistic regression	
	
	OR (95% CI)	*P*	OR_adj_ (95% CI)	*P*
Sex			0.43 (0.25–0.74)†	0.003†
Male	1			
Female	0.80 (0.60–1.05)	0.108		
Age			0.81 (0.59–1.12)	0.206
Tertile 1	1			
Tertile 2	1.09 (0.80–1.49)	0.632		
Tertile 3	1.00 (0.73–1.38)	> 0.99		
BMI			1.02 (0.75–1.40)	0.887
Normal	1			
Overweight	1.33 (0.97–1.83)	0.080		
Obese	1.31 (0.92–1.86)	0.155		
Smoking history			1.90 (1.17–3.08)†	0.009†
Never smoked	1			
Ever smoked	1.62 (1.25–2.12)	< 0.001		
Disease duration			0.64 (0.48–0.86)†	0.003†
Tertile 1	1			
Tertile 2	0.64 (0.46–0.89)	0.008		
Tertile 3	0.67 (0.48–0.93)	0.018		
Seropositivity			1.03 (0.61–1.74)	0.926
Negative	1			
Positive	1.06 (0.80–1.41)	0.717		
DAS28			3.61 (2.15–6.07)†	< 0.001†
<3.2	1			
3.2–5.19	3.46 (2.24–5.36)	< 0.001		
≥5.2	15.06 (9.31–24.36)	< 0.001		
1987 ACR criteria			1.59 (0.91–2.77)	0.103
<4 criteria	1			
≥4 criteria	2.60 (1.99–3.38)	< 0.001		
Extraarticular disease			0.79 (0.42–1.50)	0.478
No	1			
Yes	1.21 (0.85–1.73)	0.322		
Erosions			0.92 (0.55–1.55)	0.921
None	1			
Yes	0.97 (0.73–1.30)	0.881		
DAS28-P index			1.42 (1.04–1.93)†	0.026†
Tertile 1	1			
Tertile 2	2.35 (1.61–3.44)	< 0.001		
Tertile 3	4.57 (3.00–6.95)	< 0.001		
Comorbidities			1.55 (0.94–2.54)	0.086
0	1			
≥1	1.49 (1.14–1.93)	0.003		
SF-36 mental health			2.67 (1.96–3.65)†	< 0.001†
Tertile 1	1			
Tertile 2	3.09 (2.24–4.27)	< 0.001		
Tertile 3	7.89 (5.51–11.31)	< 0.001		

* Unadjusted odds ratios (ORs) and adjusted ORs (OR_adj_) with 95% confidence intervals (95% CIs) show associations at baseline between bodily pain (classified as worse or better than median bodily pain score) and classic risk factors for rheumatoid arthritis (RA) severity (n = 368). After adjustment, worse pain at baseline was associated with male sex, previous smoking, shorter disease duration, more disease activity, higher proportion of the Disease Activity Score in 28 joints (DAS28) attributable to patient-reported components (DAS28-P), and worse mental health. DAS28 groups, as classified by the European League Against Rheumatism, were 0–3.19 (inactive), 3.2–5.19 (active), and ≥5.2 (severe), and body mass index (BMI) groups were <25 kg/m^2^ (normal), 25–29.9 kg/m^2^ (overweight), and ≥30 kg/m^2^ (obese; derived from World Health Organization guidelines). Other continuous data were divided into tertiles of increasing severity for analysis. ACR = American College of Rheumatology; SF-36 = Short Form 36.

† Statistically significant data after adjustment (*P* < 0.05).

### Predictors of less improvement in pain after 1 year

The change in pain scores at 1 year was expressed as a percentage change from baseline, with a median improvement of 19.3% (IQR −16.0% to 80.5%; *P* < 0.001 versus baseline). Most patients with early RA reported incomplete improvement in bodily pain after 1 year (58%), with the remainder reporting bodily pain scores that were either the same as (15%) or worse than (27%) baseline. Statistically significant univariate associations with less pain improvement at 1 year were seen for female sex, not fulfilling the 1987 ACR criteria, seronegativity, lower bodily pain, better mental health, and lower disease activity at baseline (Table 4).

**Table 4:** Predictors of less improvement in bodily pain after 1 year*

Covariate and tertiles/groups	Unadjusted analyses		Logistic regression	
	
	OR (95% CI)	*P*	OR_adj_ (95% CI)	*P*
Sex			3.41 (1.35–8.64)†	0.010†
Male	1			
Female	1.62 (1.03–2.53)	0.041		
Age			1.42 (0.84–2.39)	0.189
Tertile 1	1			
Tertile 2	1.26 (0.76–2.09)	0.367		
Tertile 3	1.00 (0.60–1.65)	> 0.99		
BMI			1.63 (0.99–2.70)	0.055
Normal	1			
Overweight	0.79 (0.47–1.31)	0.369		
Obese	1.02 (0.58–1.79)	> 0.99		
Smoking history			0.99 (0.49–2.06)	0.999
Never smoked	1			
Ever smoked	1.03 (0.68–1.57)	0.915		
Disease duration			1.02 (0.64–1.63)	0.925
Tertile 1	1			
Tertile 2	1.19 (0.71–2.00)	0.513		
Tertile 3	1.13 (0.67–1.93)	0.684		
Seropositivity			0.61 (0.26–1.42)	0.250
Negative	1			
Positive	0.56 (0.35–0.89)	0.017		
DAS28			0.60 (0.27–1.34)	0.208
<3.2	1			
3.2–5.19	0.28 (0.13–0.58)	< 0.001		
≥5.2	0.14 (0.06–0.29)	< 0.001		
1987 ACR criteria			1.48 (0.64–3.42)	0.359
<4 criteria	1			
≥4 criteria	0.44 (0.29–0.68)	< 0.001		
Extraarticular disease			0.95 (0.36–2.52)	0.918
Normal	1			
Yes	0.75 (0.44–1.30)	0.334		
Erosions			0.95 (0.41–2.22)	0.901
None	1			
Yes	0.70 (0.43–1.12)	0.138		
DAS28-P index			2.09 (1.24–3.55)†	0.006†
Tertile 1	1			
Tertile 2	1.53 (0.86–2.70)	0.153		
Tertile 3	1.38 (0.74–2.59)	0.340		
SF-36 bodily pain			0.20 (0.10–0.38)†	< 0.001†
Tertile 1	1			
Tertile 2	0.13 (0.07–0.23)	< 0.001		
Tertile 3	0.05 (0.02–0.10)	< 0.001		
SF-36 mental health			1.08 (0.67–1.75)	0.755
Tertile 1	1			
Tertile 2	0.57 (0.35–0.94)	0.033		
Tertile 3	0.34 (0.20–0.58)	< 0.001		
Comorbidities			1.23 (0.56–2.68)	0.606
0	1			
≥1	1.27 (0.83–1.93)	0.286		
Steroids at 1 year			0.87 (0.32–2.35)	0.782
No	1			
Yes	0.85 (0.51–1.42)	0.607		
MTX at 1 year			0.71 (0.33–1.49)	0.359
No	1			
Yes	0.71 (0.47–1.08)	0.136		

* Unadjusted odds ratios (ORs) and adjusted ORs (OR_adj_) with 95% confidence intervals (95% CIs) show associations with less improvement in pain. Unadjusted ORs for pain are shown in comparison to the least severe or smallest reference group/tertile. The logistic regression model for less improvement in bodily pain score at 1 year analyzed the risks for below median change (n = 184) and compared risk increases per group/tertile. After adjustments, less improvement in pain was associated with female sex, less pain at baseline, and a higher Disease Activity Score in 28 joints (DAS28) attributable to patient-reported components (DAS28-P) index. Covariates represent baseline characteristics, except that medications (methotrexate [MTX] or sulfasalazine and steroids) at 1 year were included to address possible confounding by treatment of inflammatory disease. DAS28 groups, as classified by the European League Against Rheumatism, were 0–3.19 (inactive), 3.2–5.19 (active), and ≥5.2 (severe), and body mass index (BMI) groups were <25 kg/m^2^ (normal), 25–29.9 kg/m^2^ (overweight), and ≥30 kg/m^2^ (obese; derived from World Health Organization guidelines). Other continuous data were divided for analysis into tertiles with increasing magnitude or severity. Similar findings were obtained with another model where MTX was replaced with sulfasalazine at 1 year. ACR = American College of Rheumatology; SF-36 = Short Form 36.

† Statistically significant data after adjustment (*P* < 0.05).

Logistic regression analysis was performed to determine independent predictors of less improvement in pain at 1 year. Sixteen demographic and clinical covariates were included in the logistic regression model and are shown in Table 4. Baseline bodily pain was included to control for the regression to the mean. After adjustment, a high DAS28-P index at baseline was associated with less improvement in pain after 1 year (Table 4). Among the other variables, only female sex and low baseline bodily pain scores were associated with less improvement in pain. Inclusion of methotrexate or sulfasalazine use at 1 year in the analysis did not alter the findings from logistic regression analysis. Additionally, a smaller logistic regression model that examined only the DAS28-P and baseline pain also indicated that a high baseline DAS28-P predicted less improvement in bodily pain at 1 year (see Supplementary Appendix B, available in the online version of this article at http://onlinelibrary.wiley.com/journal/10.1002/(ISSN)2151-4658). Additionally, exclusion of the DAS28 from our model did not substantially alter the significant association between the DAS28-P and less improvement in pain (data not shown). Further analysis showed that 1-year pain levels (rather than pain change) were also associated with the baseline DAS28-P after adjustments for baseline pain and other covariates (see Supplementary Appendix B, available in the online version of this article at http://onlinelibrary.wiley.com/journal/10.1002/(ISSN)2151-4658). Additional secondary analysis of 1-year cross-sectional data showed, in contrast to the findings at baseline, that female sex was associated with greater pain (Z = −2.6, *P* = 0.009). Inclusion of NSAID use at baseline into our logistic regression model for pain change did not show a significant association and did not alter the main findings for other covariates.

## DISCUSSION

Pain is a difficult symptom to measure because it fluctuates and may be experienced or described differently by different people at different times. Over 1 year after presentation with RA, as in other studies, we found that pain levels improved, but also that pain persisted rather than completely resolved (16, 35). Logistic regression analysis found that lower baseline pain, female sex, and a high baseline DAS28-P index were associated with less pain improvement at 1 year, whereas many factors were independently associated with pain at baseline. The predictive value of the DAS28-P index may indicate that noninflammatory mechanisms contribute to the persistence of pain during the first year when the inflammatory component of the disease is treated.

The DAS28 is a validated measure of inflammatory disease activity in RA, although high DAS28 scores may sometimes be associated with central sensitization rather than active inflammation. Individual DAS28 components each increase with growing disease activity, whereas disproportionate increases in patient-reported components may be an indication of noninflammatory pain mechanisms. We calculated the DAS28-P index based on the proportion of the DAS28 that was attributable to the patient's reported joint tenderness and disease activity.

The DAS28-P index displayed validity as a measure of the patient's experience of RA, extending the information provided by the total DAS28. High DAS28-P indices were associated with worse pain both at baseline and at 1-year followup, consistent with a high contribution of pain to patients' self-reported disease activity and TJCs. The DAS28-P index displayed greater statistical independence from the total DAS28 score than did individual DAS28 components, and the DAS28-P index did not change significantly over 1 year in those whose RA remained active, despite improvements in the total DAS28 (24) and bodily pain scores. The DAS28-P acted independently of baseline bodily pain scores and the total DAS28 as a predictor of less pain improvement at 1 year. Whereas a high DAS28 indicates inflammatory disease activity, a high DAS28-P may suggest noninflammatory pain mechanisms.

Together our data indicate that the DAS28-P index represents a stable characteristic that is distinct from inflammatory disease activity in patients with continuing active disease despite conventional therapy. Factors that lead to a higher DAS28-P index appear to function before the end of the first year of RA symptoms, and a high DAS28-P index may be present at baseline and is not related to symptom duration. However, further studies would be required to determine whether the DAS28-P remains stable over longer time periods, and whether it may change in response to non-DMARD interventions.

The DAS28 is commonly used to inform treatment decisions, and health services such as those in the UK may restrict funding for biologic agents to patients with high DAS28 scores. Our data indicate that high DAS28 scores may group together patients with different disease phenotypes, and that the relative contributions made by the different DAS28 components have prognostic significance. A better understanding of the contributions of DAS28 components to treatment outcomes has the potential to enhance the equitable use of disease activity scores in treatment allocation.

Furthermore, factors that contribute to high DAS28-P indices may be targets for intervention in early RA, complementing treatments that focus on the inflammatory component of the disease. However, the DAS28-P may have limited utility in people with inactive DAS28 scores (<3.2), where small denominators used in its calculation would lead to high measurement error. Further research would be required to determine whether the DAS28-P has validity and utility in making treatment decisions.

Less intense disease-modifying treatments are sometimes offered to those people with early RA who have relatively few observed signs of inflammation (36). However, the DAS28-P index predicted poor pain outcome even when DMARD choice was included in the regression model. We hypothesize that high DAS28-P indices may identify patients with noninflammatory mechanisms underlying their pain, for whom treatments that are only directed at inflammation may lead to inadequate pain improvement. Other research tools not available in the current cohort, such as quantitative sensory testing, may help elucidate whether the DAS28-P is indeed an index of sensitization in RA.

Higher self-reported pain in RA has previously been found in women (37), and we found that women showed less improvement, although they also had less pain at baseline. This is consistent with at least 1 other study of early RA (15). These findings may indicate differences between men and women in the progression of pain during the first year of RA, although significant sex differences in pain changes were only demonstrated after adjustment for confounders.

People with worse baseline scores have greater potential to improve, and worse baseline pain scores predicted greater pain improvement at 1 year. The DAS28-P index also had predictive value. Logistic regression analyses adjusted for baseline pain score, indicating that for a given pain score, a higher DAS28-P index predicted less improvement in pain at 1 year. Sensitivity analyses demonstrated an association between the DAS28-P and less pain improvement, irrespective of sex or other variables in the logistic regression analysis.

The SF-36 bodily pain score addresses the overall experience of pain. Non-RA pain, for example, that arises from comorbidities, may influence responses to the bodily pain questions. High depression indices have been associated with greater pain in other studies of RA (38), and we found that bodily pain at baseline was associated with worse mental health scores. However, baseline mental health scores did not predict pain outcomes, suggesting that poor mental health may not be the predominant factor mediating pain or may be adequately managed in usual care. Osteoarthritis may be a confounder for pain and tenderness, and would be expected to persist despite the suppression of inflammatory disease. A more detailed analysis of comorbidities would be required in order to fully evaluate their contributions to pain prognosis in early RA, and this was beyond the scope of this study. Medication was not strongly associated with improvement in pain. More intense treatment including methotrexate may be used for patients with more painful disease, but may also reduce symptoms, thereby obscuring associations between medication use and pain. Different methods than ours may be more appropriate to determine the course of bodily pain in relation to medication usage.

People with high DAS28-P indices report more pain, more disability, and less vitality, characteristics that display similarities to people with fibromyalgia, where sensitization, TJCs, and global disease assessments are also high (9). A recent study found that 7–8% of patients with established RA satisfied criteria for fibromyalgia (13). Fibromyalgia may therefore also be viewed as a comorbidity that could predict pain outcomes. Although data were not available to classify fibromyalgia within the ERAN cohort, it is likely that a high DAS28-P index and fibromyalgia represent related constructs (9). Whereas a diagnosis of fibromyalgia implies a discrete diagnostic entity, our use of the DAS28-P index is consistent with a continuous phenotypic spectrum. Movement of individuals along that spectrum may explain why patients with RA often satisfied criteria for fibromyalgia intermittently, with almost 20% of patients with established RA fulfilling fibromyalgia criteria at some time during followup (13).

Some other limitations exist in our study. In the absence of any reference group, it is not possible to determine whether our findings are specific for RA or may be representative of the early stages of other chronic painful conditions. The ERAN recorded the onset and diagnostic codes of comorbidities, but not their severity of treatment. Data were excluded from a proportion of patients that had not filled in the SF-36 questionnaire, although the demographics of the groups analyzed remained representative of the study population. The clinical importance of the change in pain level is difficult to evaluate. In our logistic regression models we used the median improvement in pain of approximately 20% as the cutoff between more or less improvement. Small changes in pain scores may not have clinical importance for people with musculoskeletal conditions (39), and further research would be required to determine whether our “less improved” group includes people with clinically important deterioration in pain who may benefit from different treatments aimed at preventing pain deterioration.

In summary, the factors most likely to be related to poorer pain outcomes in early RA are female sex and a high baseline DAS28-P index. Noninflammatory factors such as central sensitization may contribute to poor pain prognosis in early RA. The DAS28-P index may provide an index of noninflammatory pain for epidemiologic studies in RA, and warrants further validation studies.
