# Supplementary material for: Predictors of Change in Bodily Pain in Early Rheumatoid Arthritis: An Inception Cohort Study
Source: Arthritis Care Res (Hoboken). 2012 Sep 28;64(10):1505–13. doi: 10.1002/acr.21723 (PMC3770924; doi:10.1002/acr.21723)
Supplement: Supplementary file 1 [file acr0064-1505-SD1.doc]

**Supplementary Appendix A: The effects of inactive DAS28 scores on the calculation of DAS28-P**

**Background**

We investigated the distribution of the DAS28-P formula output for all study participants, regardless of baseline DAS28.

**Methods**

The following formula was used.

(0.56 x √TJC) + (0.014 x GH)

(0.28 x √SJC) + (0.56 x √TJC) + (0.7 x LN(ESR)) + (0.014 x VAS-GH)

The formula was used for every study participant where data were available, including those presenting with inactive disease. Histograms of the results were plotted for each of the DAS28 groups based on EULAR recommendations about disease activity (<3.2-inactive; 3.2 to 5.19-active; ≥5.2-severe).

**
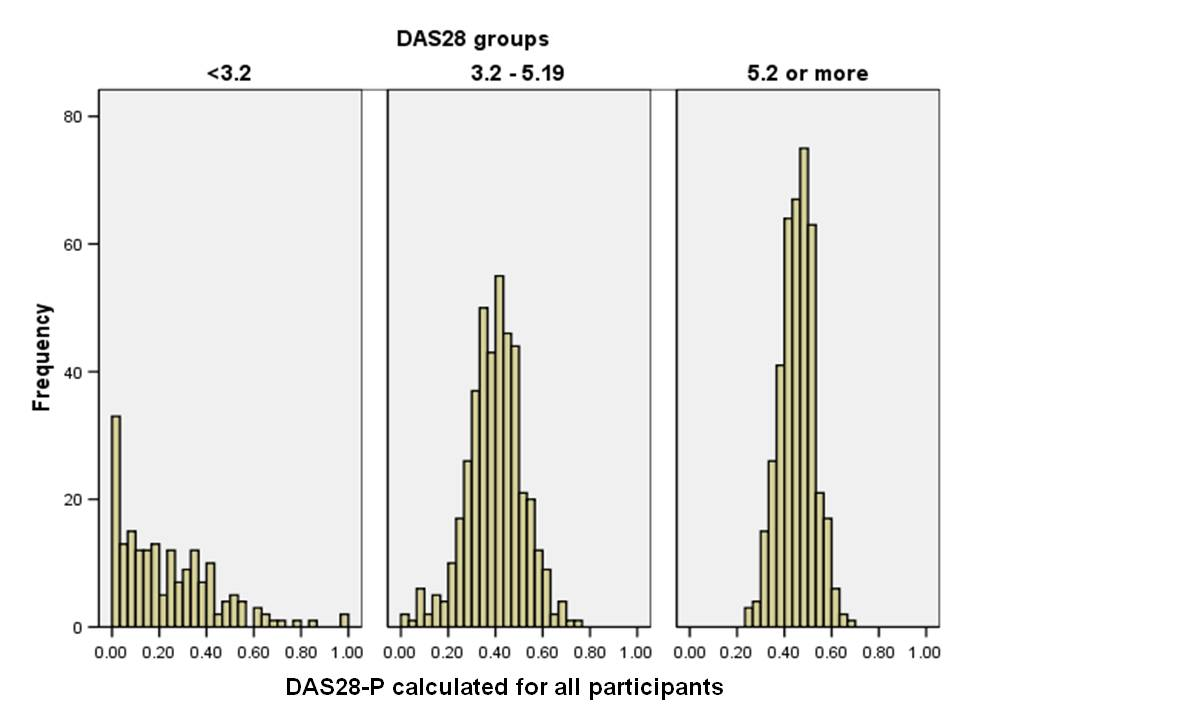
**

**Results and discussion**

The effects of inactive DAS28 scores (DAS28 < 3.2) markedly changed the output of the formula. The distribution and variability were both altered in the range DAS28 < 3.2. Within other ranges, a distribution similar to normality was observed, with less variation than the < 3.2 group. Small denominators may contribute to high variation in DAS28-P values where DAS28<3.2. Furthermore, DAS28-P as a measurement to help better understand the contribution of patient-reported components to DAS28 would have little clinical utility in patients with inactive disease. Participants with DAS28 <3.2 were therefore excluded from calculation of DAS28-P in the current study.

**Supplementary Appendix B: The association of DAS28-P with measures of pain at 1 year.**

**Background**

We evaluated two separate measures of 1 year pain scores to assess the generalisability of the relationship of pain at one year with DAS28-P.

**Methods**

Related measures of one year pain were calculated from the SF36-bodily pain data. “1 year pain” used the absolute value of SF36-bodily pain at one year. “Pain change %” normalised the Pain difference value from baseline to one year and was expressed as a % change from baseline (this is the measure used in the manuscript). Logistic regression models were gradually built to examine whether DAS28-P was associated with pain at one year when different covariates were used for adjustment (all models were adjusted for baseline SF36-bodily pain).

**Results and discussion**

High DAS28-P at baseline predicted worse pain at one year and less improvement in pain at one year. Adjustment for different covariates did not remove the relationship between DAS28-P and one year pain.

| **Logistic regression covariates** | **1 year pain** | |  | | **Pain change %** | | | | |  | | |
| --- | --- | --- | --- | --- | --- | --- | --- | --- | --- | --- | --- | --- |
|  | **aOR (95% CI)** | **p** |  | | **aOR (95% CI)** | | **p** | | |  | | |
|  |  |  |  | |  | |  | | |  | | |
| **DAS28-P + Pain** | 1.60 (1.16 - 2.19) | 0.004 |  | | 1.79 (1.22 - 2.62) | | 0.003 | | |  | | |
|  |  |  |  | |  | |  | | |  | | |
| **DAS28-P + Pain + Age + Gender** | 1.61 (1.15 - 2.24) | 0.005 |  | | 1.97 (1.31 - 2.95) | | 0.001 | | |  | | |
|  |  |  |  | |  | |  | | |  | | |
| **DAS28-P + Pain + Age + Gender + DAS28** | 1.64 (1.16 - 2.35) | 0.006 |  | | 2.03 (1.33 - 3.09) | | 0.001 | | |  | | |
|  |  |  |  | |  | |  | | |  | | |
| **Full model** | 1.66 (1.03 - 2.67) | 0.036 |  | | 2.09 (1.24 - 3.55) | | 0.006 | | |  | | |
| Logistic regression models with increasing numbers of covariates,  showing the associations between DAS28-P and pain | | | | | | | | | | | | |
| Risks for worse than median pain at 1yr and less improvement at  1 year (% difference) than median are shown | | | | | | | | | | | |  |
| Full model used the same covariates as in the main manuscript | |  | |  | |  | |  |  | |  |  |
|  | |  | |  | |  | |  |  | |  |  |
